# Could real-time sonoelastography-measured placental strain ratio (PSR) value be a soft marker for the diagnosis of intrahepatic cholestasis of pregnancy?: A case-control study and short reviews

**DOI:** 10.1097/MD.0000000000034111

**Published:** 2023-07-07

**Authors:** Halime Şen Selim, Mustafa Şengül

**Affiliations:** a Izmir Katip Celebi University Atatürk Training and Research Hospital, Obstetrics and Gynecology Department, Karabağlar/İzmir, Turkey; b Izmir Katip Celebi University, Obstetrics, and Gynecology Department, Karabağlar/İzmir, Turkey.

**Keywords:** bile acids, intrahepatic cholestasis of pregnancy, sonoelastography, strain ratio

## Abstract

Diagnosis of intrahepatic cholestasis of pregnancy (ICP) is often decided upon with typical pruritus supported by elevated serum bile acid levels. However, there is confusion regarding the absolute reference range for serum bile acid^.^ To confirm the utility of Placental Strain Ratio (PSR) measurement as a marker for the diagnosis of ICP and to reveal the extent to which it is correlated with serum bile acid concentration. A case-control study was conducted. The case group included 29 patients who were admitted to our hospital in the second or third trimester of pregnancy with typical itching and were clinically diagnosed with ICP with >10 mmol/L serum bile acid. The first 45 pregnant women were assigned to a control group. Real-time tissue elastography software was used for ultrasound assessment of all pregnant placentas. Software was used to estimate the SR values. Biochemical liver function parameters, hemograms, serum bile acid levels, and SR values were compared between these groups. PSR was found to predict the development of cholestasis with poor discrimination (area under the curve [AUC] = 0.524; 95% CI = 0.399–0.646). The optimal threshold value with the best sensitivity and specificity rates was calculated to be 0.46 PSR. ICP developed significantly more frequently in the low PSR group than in the high PSR group (60% vs 29.3%, *P* = .05, odds ratios [OR] = 0.276, 95% CI = 0.069–1.105). No correlation was found between the PSR and bile acid levels (rho = −0.029, *P* = .816). PSR values can support the diagnosis of ICP, predict serum bile acid levels, and can be used as soft markers.

## 1. Introduction

Intrahepatic cholestasis of pregnancy (ICP) is a complication that presents with classic symptoms such as pruritus, typically on the palms and planta, due to increased bile acids regardless of elevated liver function tests, generally in the late second or third trimester.^[1,2]^ ICP is the most prevalent pregnancy-specific liver disease.^[3]^ On the other hand, ICP incidence differs by ethnicity and geography and is often reported to be between 0.2% and 2%.^[1]^ Risk factors for ICP include family history, advanced maternal age, multiple pregnancies, history of liver-related diseases (e.g., gallstones or hepatitis C), and other concomitant pregnancy-related disorders (e.g., gestational diabetes and preeclampsia)^[4–6]^

The diagnosis of ICP is often based on typical pruritus supported by elevated serum bile acid levels. However, there is confusion regarding the absolute reference range of serum bile acid^[7]^ because general reference ranges proposed for mixed male-female groups are used instead of the trimester-specific reference range for pregnant women.^[7–9]^ Moreover, while direct spectrophotometry adopts the average bile acid concentration as 1.8 _ 2.2 mmol/L (range 0.5–12.0), enzymatic methods consider it to be 6.5 mmol/L (2 SD range 1.7–11.3)^[10,11]^ In addition, laboratory abnormalities often fall behind the clinic, creating complexity in diagnosis, and the guidelines-literature gap confirms the difficulty of clinical diagnosis and management.^[12]^ Therefore, introducing a novel marker to support the diagnosis of cholestasis or to predict bile acid levels may help overcome the difficulties mentioned above. In addition, the accumulation of high bile acids in the placental bed is known to cause fetal morbidity.^[13]^ Although it is most likely to identify such changes in the placenta pathologically,^[14]^ the examination seems impossible during pregnancy. In this sense, detecting placental changes using noninvasive measurements may also aid in diagnosis.

Modern perinatal medicine uses different ultrasound investigations of the placenta to detect various diseases and conditions in pregnancy, such as fetal macrosomia and gestational diabetes^[15,16]^

Expanded placental villis and destroyed villi structure in ICP^[17]^ and finding of enlarged anterior placenta with cystic spaces on ultrasound exam^[18]^ provide the rationale for further ultrasonographic evaluation of placenta in management and diagnosis of ICP. One of the various ultrasound investigations available is sonoelastography (SE); this method has the potential to evaluate the accumulation of high levels of bile acids in the placental bed^[13]^ of women with ICP.

SE is a functional measurement to demonstrate tissue rigidity by ultrasound.^[19]^ Although SE was initially deployed to detect the differentiation of malignant and benign tissues, recent scholarly work has confirmed its safety for pregnant women and considered it valuable in evaluating placental functions.^[20–22]^

In this study, we investigated whether the real-time SE-measured placental strain ratio (PSR) reflects bile acid accumulation in the placenta of patients diagnosed with intrahepatic cholestasis. Accordingly, we aimed to confirm the usability of PSR measurement as a marker for ICP diagnosis and to reveal the extent to which it is correlated with serum bile acid concentration.

## 2. Materials and methods

This case-control study was conducted between August 1, 2021, and September 30, 2022, after the Ethics Committee of XXX Training and Research Hospital—XXX University granted ethical approval for our study (No. 63). The study complied with the principles of the Declaration of Helsinki. Signed consent forms were obtained from all patients.

The case group included 29 patients who were admitted to our hospital in the second or third trimester of pregnancy with typical itching and were clinically diagnosed with ICP with >10 mmol/L serum bile acid. We then included the first 45 pregnant women with similar characteristics to the case group (age, gravida, parity, gestational week), but without any itching complaints or any pregnancy complications (e.g., gestational diabetes mellitus, hypertension, intrauterine growth retardation) in the control group. We followed all participants until birth and noted down delivery type, birth weight, appearance, pulse, grimace, activity, and respiration score (APGAR)-1 and APGAR-5, intrauterine growth restriction (IUGR), and neonatal intensive care unit (NICU) need parameters.

Blood samples were obtained to evaluate routine biochemical parameters (aspartate aminotransferase [AST], alanine aminotransferase [ALT], Bilirubin levels), hemograms, and serum bile acid levels. We also measured the patients’ blood pressure and excluded those with a diastolic blood pressure ≥90 mm Hg or systolic blood pressure ≥140 mm Hg. Moreover, 6 pregnant women in the case group - 3 who did not accept PSE, 2 with accompanying preeclampsia, and one with a suspected scabies lesion - were excluded from the study.

**Figure 1.**
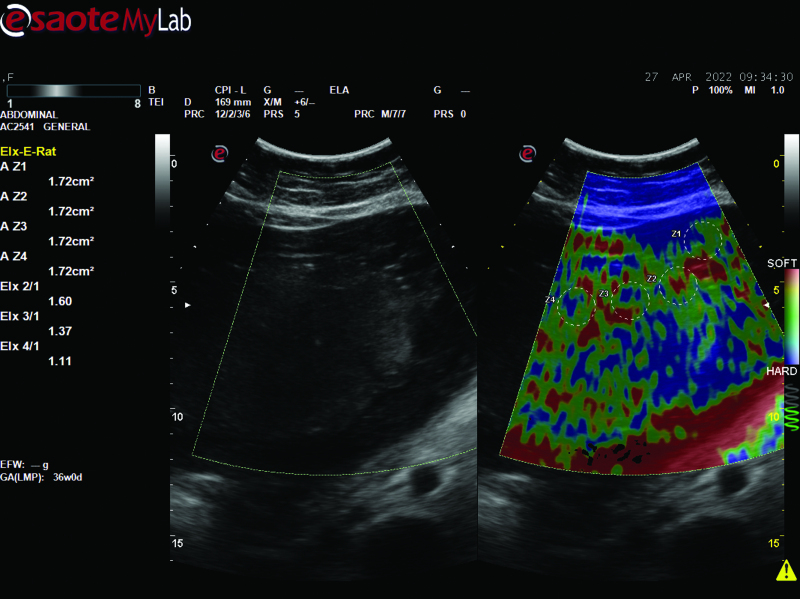
The dual screen of the B-mode view next to the elastography. On this screen, 1 region of interest (ROI) was placed on the myometrium [A, (Z1 circle)] and 3 ROI [B, (Z2, Z3, and Z4 circles)] on the placenta, and each ROI area (Z1, Z2, Z3, and Z4) was measured to be 0.76 cm^2^. The software estimated strain ratio (SR) values (Elx2/Elx1, Elx3/Elx1, and Elx4/Elx1) using these 4 ROI values (Elx1, Elx2, Elx3, and Elx4).

We used Real-time tissue elastography software (Esaote MyLabSeven, Esaote Group, Genova, Italy) and an 8-1 MHz multi-frequency AC2541 probe were used for ultrasound assessments of all patients with an empty bladder and breath-hold. The probe was compressed and decompressed and tissue elasticity was measured using a transducer based on color codes. Red indicates soft tissue, whereas blue indicates hard tissue. Next, proper compression was decided on to 4 to 7 boxes of the scale, with 7 boxes turning green. We then evaluated placental elasticity on the dual screen in the B-mode view, followed by elastography. On this screen, 1 region of interest (ROI) was placed on the myometrium [A, (Z1 circle)] and 3 ROI [B, (Z2, Z3, and Z4 circles)] were placed on the placenta. Each ROI area (Z1, Z2, Z3, and Z4) was measured to be 0.76 cm^2^. The software-estimated strain ratio (SR) values (Elx2/Elx1, Elx3/Elx1, and Elx4/Elx1) using these 4 ROI values (Elx1, Elx2, Elx3, and Elx4) (are shown in Fig. 1). We described the mean strain ratio as PSR [(SR1 + SR2 + SR3)/3] in our study, as in the study by Eroğlu et al^[23]^ The PSR values of pregnant women in both the case and control groups were recorded and compared. In addition, the reliability of the study was promoted because the same researcher (XXX XXX) performed the real-time SE measurements.

### 2.1 Statistical analysis

Continuous variables were presented as mean, minimum, and maximum values, while categorical variables were presented as percentages (%). Kolmogorov-Smirnov analysis was used to check the normality of the distribution, we used Kolmogorov-Smirnov analysis. Accordingly, for normally distributed data, we performed an independent samples *t* test for pairwise comparisons and Pearson chi-square test (Fisher exact test in the case of a small sample size [n ≤ 5]) to compare categorical data. For non-normally distributed data, pairwise comparisons were performed using the Mann–Whitney *U* test. Interquartile ranges were also provided for the median values of the variables. We then recruited variables that were determined to affect cholestasis in the univariate analysis for multivariate analysis to identify independent risk factors affecting cholestasis. Moreover, we constructed a receiver operating characteristic (ROC) curve to explore the predictors (s) of cholestasis formation using placental elastography thickness and calculated the area under the curve (AUC). ROC analysis yielded the best sensitivity and specificity percentages to determine the “optimal” cutoff point for placental elastography values for cholestasis. We then calculated the negative and positive predictive values for this threshold value, which led to grouping the patients into 2 groups as those with values below and above the threshold value. Finally, we compared the groups based on cholestasis and calculated odds ratios (OR). We also calculated Spearman rank correlation coefficient (rho) to reveal the relationship between placental elastography and bile acid values. As a rule of thumb, the correlations denoted by rho values were grouped as very weak (<0.2), soft (0.2–0.4), moderate (0.4–0.6), and high (>0.6). All analyses were performed using the Statistical Package for the Social Sciences (IBM SPSS Statistics for Windows, Version 23.0, Armonk, NY); a *P* value < .05 to was considered statistically significant.

## 3. Results

Table 1 presents the patients’ demographic characteristics, follow-up findings, and newborn findings.

**Table 1:** Patients’ demographic characteristics and follow-up findings during pregnancy and the findings of their newborns.

Variables	
*Demographic characteristics*	
Age, mean (SD)	28.7 (6.0)
Gravida, median (IQR)	2 (2)
Parity, median (IQR)	1 (2)
BMI, kg/m^2^ mean (SD)	28.6 (4.5)
Comorbidity, n (%)	18 (26.5)
Previous cholestasis history, n (%)*	6 (13.0)
Previous cesarian section, n (%)*	18 (39.1)
*Pregnancy follow-up variables*	
PSR (IQR)	0.79 (0.47)
AST, (IU/L) median (IQR)	20.0 (12.0)
ALT, (IU/L) median (IQR)	14.0 (16.8)
GGT, (U/L) median (IQR)	45.0 (42.0)
LDH, (U/L) median (IQR)	101.5 (31.0)
Bile acid (mmol/L), median (IQR)	0.0 (11.0)
Total bilirubin (mg/dL), median (IQR)	0.38 (0.25)
Direct bilirubin (mg/dL), median (IQR)	0.09 (0.10)
Indirect bilirubin (mg/dL), median (IQR)	0.30 (0.23)
*Postnatal variables*	
Type of delivery, n (%)	
Normal	16 (23.5)
C-section	52 (76.5)
Birth weight (g), median (IQR)	3167 (395)
Apgar 1, median (IQR)	9 (0)
Apgar 5, median (IQR)	10 (0)
Newborn sex, n (%)	
Female	45 (66.2)
Male	23 (33.8)
Intrauterine growth retardation, n (%)	1 (1.5)
NICU need, n (%)	1 (1.5)

ALT = alanine aminotransferase, APGAR = appearance, pulse, grimace, activity, and respiration, AST = aspartate aminotransferase, BMI = body mass index, IQR = interquartile range; LDH = lactate dehydrogenase, n = number, NICU = neonatal intensive care unit, PSR = placental strain ratio.

* Calculations were performed using data from 42 patients.

It was discovered that 23 patients (33.8%) developed cholestasis during the follow-up period. On the other hand, Table 2 comparatively shows the demographic and follow-up data of the case (n = 23) and control groups (n = 45). Accordingly, the findings showed no significant differences between the groups in terms of age (*P* = .640), gravidity (*P* = .393), parity (*P* = .538), body mass index (*P* = .990), and PSR (*P* = .751). Moreover, we found that a previous history of cholestasis was significantly higher in those who developed cholestasis than in those who did not (35.3% vs 0%, *P* = .002). In addition, male sex was significantly higher among newborns of patients with cholestasis (56.5% vs 22.2%, *P* = .005). Multivariate analysis also showed that cholestasis was an independent risk factor for cholestasis to be the previous history of cholestasis (OR = 31.224, 95% CI = 2.855–341.451, *P* = .005) and sex of the newborn (OR = 8.896, 95% CI = 2.351–33.656, *P* = .001; Table 2).

**Table 2:** Comparison of the patients with (n = 23) and without (n = 45) cholestasis.

Variables	Patients without cholestasis (n = 45)	Patients with cholestasis (n = 23)	Univariate	Multivariate		
			*P* value	OR	95% CI	*P* value
Age, mean (SD)	29.0 (6.0)	28.2 (9.0)	.640	0.911	0.804–1.032	.142
Gravida, median (IQR)	2 (2)	2 (2)	.393	4.069	0.226–73.193	.341
Parity, median (IQR)	1 (2)	1 (2)	.538	0.352	0.020–6.034	.471
BMI, kg/m^2^ mean (SD)	28.6 (4.4)	28.6 (7.8)	.990	1.048	0.915–1.200	.502
Previous cholestasis history, n (%)	-- (0)	6 (35.3)	**.002**	31.224	2.855–341.451	**.005**
PSR, median (IQR)	0.79 (0.44)	0.80 (0.56)	0.751	1.113	0.213–5.821	.899
Newborn sex, n (%)			**.005**	8.896	2.351–33.656	**.001**
Female	35 (77.8)	10 (43.5)				
Male	10 (22.2)	13 (56.5)				

BMI = body mass index, IQR, interquartile range; n, number, OR = odds ratios, PSR = Placental Strain Ratio.

The *P* values in bold indicate statistical significance, while the *P* values in italics indicate almost significant relationships.

The results of the liver function tests are presented in Table 3. Accordingly, the AST (*P* < .001), ALT (*P* < .001), lactate dehydrogenase (*P* < .001), total bilirubin (*P* < .001), direct bilirubin (*P* < .001), and indirect bilirubin (*P* = .01) levels were significantly higher in the case group than in the control group. The clinical status of the newborns and postnatal findings in the case and control groups are shown in Table 3. In this regard, we did not find any significant differences between the groups according to delivery type, birth weight, APGAR-1 and APGAR-5, IUGR, or NICU needs.

**Table 3:** Comparison of the patients with and without cholestasis by liver function tests, postnatal findings and clinical status of their newborns.

Variables	Patients without cholestasis (n = 45)	Patients with cholestasis (n = 23)	*P* value
AST, (IU/L) median (IQR)	17 (9)	36 (46)	**<.001**
ALT, (IU/L) median (IQR)	11 (7)	30 (62)	**<.001**
GGT, (U/L) median (IQR)	65 (37)	41 (24)	**<.001**
LDH, (U/L) median (IQR)	98 (10)	150 (35)	**<.001**
Total bilirubin, (mg/dL), median (IQR)	0.33 (0.19)	0.49 (0.18)	**<.001**
Direct bilirubin, (mg/dL), median (IQR)	0.07 (0.05)	0.12 (0.10)	**<.001**
Type of delivery, n (%)			.394
Normal	12 (26.7)	4 (17.4)	
C-section	33 (73.3)	19 (82.6)	
Birth weight, g, median (IQR)	3180 (380)	3160 (495)	.995
Apgar 1, median (IQR)	9 (0)	9 (0)	.589
Apgar 5, median (IQR)	10 (0)	10 (0)	.906
Intrauterine growth retardation, n (%)	--	1 (4.3)	.338
NICU need, n (%)	--	1 (4.3)	.338

ALT = alanine aminotransferase, APGAR = appearance, pulse, grimace, activity, and respiration, AST = aspartate aminotransferase, IQR: interquartile range, LDH = lactate dehydrogenase, n = number, NICU = neonatal intensive care unit.

The *P* values in bold indicate statistical significance.

**Figure 2.**
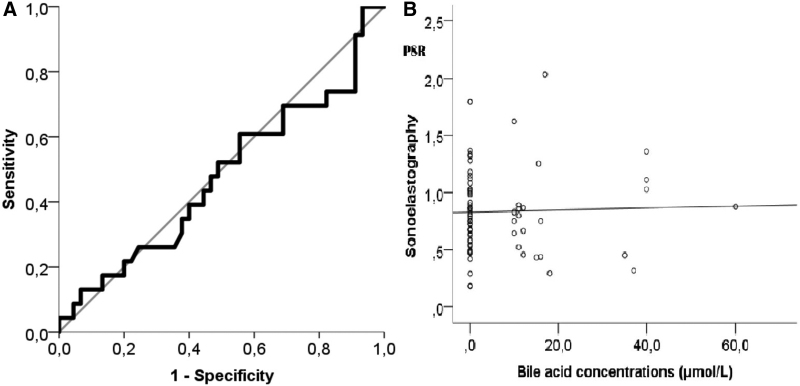
(A) ROC curve analysis-PSR was found to predict the development of cholestasis at poor discrimination (AUC = 0.524; 95% CI = 0.399–0.646). (B) Correlation between PSR and bile acid level (rho = -0.029, *P* = .816). AUC = area under the curve, PSR = Placental Strain Ratio, ROC = receiver operating characteristic.

In the ROC curve analysis, PSR was found to predict the development of cholestasis with poor discrimination (AUC = 0.524; 95% CI = 0.399–0.646; Fig. 2A). The optimal threshold value with the best sensitivity and specificity rates was calculated to be 0.46 PSR. Therefore, the patients were divided into 2 groups according to the threshold value: the low PSR group (n = 10) and the high PSR group (n = 58). ICP developed significantly more frequently in the low PSR group than in the high PSR group (60% vs 29.3%, *P* = .05, OR = 0.276, 95% CI = 0.069–1.105; Table 4). Finally, we found no correlation between PSR and bile acid levels (rho = -0.029, *P* = .816; Fig. 2B).

**Table 4:** The findings of placental elastography thickness predicting cholestasis and the distribution of the patients with and without cholestasis by determined cutoff value.

	AUC	95%CI	cutoff	Sensitivity (%)	Specificity (%)	PPV (%)		NPV (%)
Placental elastography thickness	0.524	0.399–0.646	0.46	26.0	91.1	60.0		70.7
Cutoff values	Total (n)	Patients without cholestasis (n = 45)		Patients with cholestasis (n = 23)		*P*	OR	95%CI
Cutoff ≤ 0.46, n (%)	10	4 (40.0)		6 (60.0)		*.05*	0.276	0.069–1.105
Cutoff > 0.46, n (%)	58	41 (70.7)		17 (29.3)				

AUC = area under the curve, CI = confidence interval, NPV = negative predictive value, n = number, OR = odds ratio, PPV = positive predictive value.

*P* values in italics indicate significant relationships.

## 4. Discussion

Diagnosis of ICP is considered critical because it is the most prevalent pregnancy-specific liver disease^[3]^ and causes several undesirable fetal outcomes, such as preterm labor, fetal asphyxia, and intrauterine death.^[24]^ The diagnosis is often decided based on an elevated serum bile acid level, as well as the characteristic complaints of pruritus; however, there is much confusion about the reference serum bile acid level in pregnancy. For example, a study investigating normal bile acids levels among 219 healthy women with a singleton pregnancy reported that the measurements of 216 women ranged between 0.3 and 9.8 μmol/L, with 3 measurements outside this range.^[25]^ Moreover, clinical symptoms sometimes emerge nearly 3 weeks before the bile acid level increases. Therefore, if patients have an average serum bile acid level and complain of typical itching, they are recommended to have serum bile acid concentrations measured every 1 to 2 weeks.^[1,26]^ In addition, some countries (e.g., Turkey) may offer limited access to laboratory measurements of bile acid levels.

Increased bile acids in ICP appear to be responsible for neonatal complications, where they accumulate in the placenta and impair placental function.^[13]^ In this sense, being able to uncover this accumulation in the placenta with a noninvasive method may provide supporting evidence or a soft marker in the diagnosis of ICP. Recent studies, most of which concentrated on preeclampsia,^[27–31]^ have suggested that SE can quantitatively assess placental function.^[27]^ When searching the expression “sonoelastography and intrahepatic cholestasis of pregnancy” on PubMed, we discovered that only 1 study investigated maternal liver elasticity by acoustic radiation force impulse (ARFI) elastosonography in ICP^[32]^ and that others are not related neither ICP nor elastosonography.^[33]^ Thus, to our knowledge, the present study is the first to explore the relationship between SE findings and intrahepatic cholestasis during pregnancy.

Many risk factors for ICP have been described, such as ethnicity, winter months in some countries (e.g., Sweden, Finland, Chile), family history of biliary disease, hepatitis C, prior ICP, multifetal gestation, in vitro fertilization, and advanced maternal age.^[3,34–37]^ Similarly, in our study, a history of cholestasis in a previous pregnancy was found to be a significant risk factor (35.3% vs 0%, *P* = .002). In a study evaluating the role of fetal sex in 2289 documented ICP pregnancies, the number of male fetuses was higher than that of female fetuses despite being statistically insignificant, which correlates with our findings.^[38]^ In our study, male sex was found to be a significant risk factor (56.5% vs 22.2%, *P* = .005).

Cytotoxicity may occur, and serum aminotransferases are elevated in approximately 60% to 85% of pregnant women with ICP because of bile acid collection in hepatocytes.^[39]^ In our study, we found that impaired liver enzymes specified in the ICP diagnostic criteria in many studies^[12,40]^ were significantly higher in the case group (AST (*P* < .001), ALT (*P* < .001), lactate dehydrogenase (*P* < .001), total bilirubin (*P* < .001), direct bilirubin (*P* < .001), and indirect bilirubin (*P* = .01)).

ICP carries numerous risks to newborns because of the possibility of being transmitted by the placenta of maternal serum bile acid to the fetus and amniotic fluid. Consequently, intrauterine fetal death, preterm delivery, meconium-stained amniotic fluid, and neonatal respiratory distress syndrome may occur.^[5,41,42]^ However, we concluded that there was no significant difference between the groups according to delivery type, birth weight, APGAR-1 and APGAR-5 levels, IUGR, and NICU needs, which again emphasizes the importance of early diagnosis and management of cholestasis.

Similar to our study, a cohort study addressing fetal outcomes of ICP pregnancies concluded that there was no significant increase in the risk of stillbirths (aOR = 0.92, 95% CI 0.52–1.62), neonatal death (aOR = 0.45, 95% CI 0.15–1.40), and meconium aspiration (aOR = 1.41, 95% CI 0.72–2.72). However, the study reported low Apgar scores (<7) at 5 minutes (aOR = 1.45, 95% CI 1.14–1.85) and more often LGA infants (aOR = 2.27, 95% CI 2.02–2.55).^[43]^

A review of 28 studies on the use of elastography in placental research reported that ultrasound elastography can be used to quantitatively evaluate the biomechanical characteristics of the placenta when placental function is restricted.^[27]^ Similarly, we found that PSR can predict the development of cholestasis (AUC = 0.524, 95% CI 0.399–0.646); poor discrimination in this study may be attributed to the small number of patients. In addition, a study evaluating placental elastography of 111 pregnant women found the placental index to be significantly higher in the group with SGA babies compared to normal babies (44.3 (± 29.4) vs 8.8 (± 10.0); *P* < .01).^[44]^

While a limited number of studies on placental elastography have mostly focused on preeclampsia and IUGR, the only elastography study on ICP has been performed using with liver elastography. In a study comparing 22 women with ICP and 33 healthy pregnant women, ARFI scores (median ARFI-R, ARFI-L, and mean ARFI) were higher in pregnant women with ICP (*P* = .015, *P* = .011, and *P* = .004, respectively). In addition, maternal liver enzyme levels and ARFI elastography scores (*R* = 0.404, *P* = .002 and *R* = 0.389, *P* = .003, respectively) were positively correlated. Finally, the optimal cutoff point of the mean maternal liver ARFI elastography score (for identifying the risk of ICP) was found to be 1.23 m/s.^[32]^

We calculated the optimal threshold PSR value as 0.46. Upon regrouping the patients with low PSR and high PSR, we found that ICP developed significantly more in the high PSR group (60% vs 29.3%, *P* = .05, OR = 0.276, 95% CI 0.069–1.105), which may also suggest that PSR can be valuable for the diagnosis of ICP.

Finally, we sought to determine the relationship between PSR and bile acid levels but did not find any significant correlation between these variables (rho = −0.029, *P* = .816), which may be due to the relatively low serum bile acid levels among our patients. Only 4 of our patients had serum bile acid levels >40 µmol/L, defined as severe cholestasis, while others had 10 to 40 micromol/L serum bile acid levels. Further studies should recruit a group of pregnant women with severe cholestasis and, thus, provide comprehensive results on the effect of serum bile acid on PSR. In addition, some results may fall into the poor discrimination zone because the limited number of patients restricted our statistical findings. In conclusion, the small number of patients with severe cholestasis was one of the limitations of our study.

On the other hand, we know that Shear-Wave elastography, a type of elastography, is less dependent on professional knowledge,^[45]^ maybe it could be a weakness of our study. However, we could not find any studies on the diagnostic effectiveness of shear wave elastography for placental function. Generally, they focused on malignancy, mostly other systems, and fewer gynecological issues, commonly in endometrial cancer^[46]^ and pelvic floor.^[47]^ In this sense, performing our study with strain elastography instead of shear-wave elastography can be considered as the second limitation of our study. However, our study will also shed light on shear wave studies related to the placenta in pregnant women with ICP.

Due to the relatively low incidence of ICP in pregnancy,^[1]^ our sample size could have not been large, which is another limitation of our study.

In this study, we showed, for the first time, the changes in placental elastography indices in pregnant women with ICP. Further research could obtain more significant results in a more extensive patient series on pregnant women with severe cholestasis and maybe by using share wave elastography. Overall, PSR values can support the diagnosis of ICP, predict serum bile acid levels, and can be used as a soft marker.

## Author contributions

**Conceptualization:** Halime Sen Selim, Mustafa Sengul.

**Data curation:** Halime Sen Selim, Mustafa Sengul.

**Formal analysis:** Halime Sen Selim.

**Investigation:** Halime Sen Selim, Mustafa Sengul.

**Methodology:** Halime Sen Selim.

**Project administration:** Halime Sen Selim.

**Resources:** Mustafa Sengul.

**Supervision:** Mustafa Sengul.

**Validation:** Halime Sen Selim, Mustafa Sengul.

**Writing – original draft:** Halime Sen Selim.

**Writing – review & editing:** Halime Sen Selim.
